# Exsudative Diathesis—A Review

**Published:** 1916-07

**Authors:** Carl G. Leo-Wolf

**Affiliations:** Buffalo, N. Y.


### Exsudative Diathesis—A Review

By CARL G. LEO-WOLF, M. D., of Buffalo, N. Y.

(Continued from June issue.)

### Therapy:

**Feeding:** Czerny (15) sums up his views on the connection between exsudative diathesis and feeding as follows: The idea that milk and eggs are the best food for children is erroneous, and is most likely a relic of the days when rickets and scrophulosis were regarded as diseases due to an insufficient supply of protein in the food. To make the general statement that woman's milk is the best food for infants is also wrong. We can only assert that the mortality amongst children at the breast is lowest. Many children do not thrive at the breast and show all kinds of symptoms while on this food, these disturbances are due to constitutional abnormalities, of which exsudative diathesis is one. Thus for instance seborrhoea faciei is due to the feeding which brings out the exsudative diathesis, and one of the symptoms of this is seborrhoea.

The growing plant at first needs only water, as it can live and grow on the material contained in the seed; later however it will need other food. The same is true of the infant, which can live on mother's milk, which is poor in building-material, up to nine months, provided it is normal and has enough building-material in its body. If it should be abnormal, however, it will suffer from diseases due to faulty metabolism, such as rachitis, anemia, status lymphaticus, spasmophilia or exsudative diathesis.

The first two years of life are of the greatest importance as concerns the appearance of exsudative diathesis.

Measles and florid tuberculosis bring out the symptoms of latent exsudative diathesis, but by proper feeding we can make the symptoms of it disappear even in these cases, because diet can control exsudative diathesis just as well as it can diabetes.

Czerny (12) further states that three points are important in the control of exsudative diathesis, namely: 1. The kind of feeding, 2. The condition of the nervous system, 3. Intercurrent infections. By treating this trio we can reduce the symptoms of exsudative diathesis to a minimum, though we cannot cure these altogether. As to the feeding, every kind of feeding which precludes fattening improves the condition of the child. The fattening may be due to 1. Excessive amounts of proper food, 2. Poor quality of the food, 3. Both. The physician must therefore ascertain first of all in every case, what the child has eaten since its birth, in order to be successful.

In children after the second year of life the diet should be principally vegetarian, with only small amounts of milk, not more than one-quarter to one-half litre of milk a day. Eggs are the worst of foods for exsudative children; cream, butter and sugar are also bad and should be allowed in small amounts only.

The feeding during the first two years of life is more difficult, because then milk is the principal diet, and the physician must so guide the feeding that the child may get along with minimal amounts of milk. The general appearance of the child and not the scales should determine the mode of feeding in every case. After the sixth month, especially in fat children, soups and vegetables are given.

Feer (20) believes that the congenital disposition and the mode of feeding should be considered as the causes of exsudative diathesis. To fight the disposition these children must be fed sparsely. They must not get milk, butter, eggs, nor meat; they should have little sugar; they are allowed large amounts of fruit and vegetables.

Simpson (84) on the other hand denies the harmful effect of milk in infants with eczema, and he does not want to change the diet of the infant as long as it is thriving.

Finkelstein (22) is of the opinion that the relations between eczema and the mode of feeding and the net gain in weight are by no means simple. Only the overfed infant may show an improvement of its eczema with reduced feeding, though this will not be necessarily so.

Steinitz (88) in his report from the Vegetarian Children's Home in Breslau, attributes his good results in keeping down the manifestations of exsudative diathesis to the diet. He is, however, convinced that not the vegetarianism was the principal therapeutic factor, but the avoidance of all fattening.

Variot (94) asserts that eczema is due to the ''Eczematigenous'' qualities of mother's milk, and that it may be cured by a change of the wet-nurse or of the food. Ibrahim, who reviews this paper, asks, however, why this milk was not tried on other babies who did not have exsudative diathesis?

Czerny (16) cites a very interesting experiment, which also proved to him that the clinical picture, which was formerly called scrophulosis, is a combination of the symptoms of tuberculosis and of exsudative diathesis.

On the advice of a visiting colleague the children in his tuberculosis ward were given all the cod liver oil they could digest; no change in the weight-curve was apparent from this, but one after another of these children developed eczema of the face or scalp, and after a few weeks the tuberculosis ward was a typical scrophulosis ward, whilst before this by proper feeding all the symptoms of exsudative diathesis had been avoided in his little tuberculous patients. After discontinuing the administration of the cod liver oil the scrophlosis disappeared again within a few weeks.

**Climate:** v. Planta (69) believes that the lessened amount of oxygen in the mountain air causes a change in the metabolism. By this the exsudative diathesis and not the asthma are to be cured. He therefore advises a long-continued sojourn, of not less than one year, as otherwise the asthma may return. This climatic treatment should be carried out in an institution, so that the diet can be watched and strictly regulated. He has come to these conclusions from his observation of the fact that mountaineers have less constitutional abnormalities and then in a milder form.

Bockhorn (8) advises a sojourn of three months at the sea-shore for children with exsudative diathesis, because the treatment of this means the prevention of tuberculosis.

**Medicines:** Krasnogorski (41) proclaims himself an adherent to the theory of Eppinger & Hess, who attribute some of the symptoms of exsudative diathesis to vagotony, an over-irritability of the autonomous innervation. He therefore recommends the administration of atropin in increasing doses. He found that the atropin was born well by the children, who could take the adult dose without any symptoms of poisoning. He claims that this drug exerts a favorable influence, in all cases, upon the exsudative symptoms, such as wheeping eczema, but especially chronic exsudative bronchitis.

Schreiber (82) has seen good results from alkaline mineral waters, also arsenic periodically, and calcium glycero-phosphate, together with a sojourn at the sea-shore. He urges great care in the administration of iodine in these cases.

**Conclusions:** I realize fully that the literature which I was able to collect on this subject, is by no means complete, as I could consider only that within my reach. I must therefore ask the indulgence of those authors whom I have inadvertently overlooked.

I have let the different authors propound their views for themselves, without any comment on my part, because I wanted to write a review on this subject rather than a monograph.

I have endeavored to avoid repetition, though this was not always possible, as I did not want to pick the different papers, which sometimes covered the whole range of our topic, to pieces any more than I could help doing.

Complicated though this subject may still appear at the first glance, I trust that the following points will come out saliently:

1. That the old theory of the crases had to be revived, in order to explain successfully some of the morbid phenomena.
2. That different diatheses will be encountered, of which exsudative diathesis stands out as a clear entity.
3. That exsudative diathesis is a constitutional abnormality which is usually inherited, and that it is the result of a disturbance of the metabolism.
4. That it appears in the different organs of the body, where it is clearly manifested.
5. That it will modify the course and appearance of other diseases, especially those of an infectious nature.
6. That its therapy is principally dietetic. That climatic changes may also have a beneficial effect, but that medicine is of minor importance in its treatment.

Finally that we must never lose sight of the fact in medical practice that we are taking care of patients and not of cases, and that this factor is the "x" of the equation.

**Bibliography:**

1. Aschenheim, E.: Eosinophilie und exsudative Diathese, Jahrbuch für Kinderheilkunde, 1912, volume 76, page 456.
2. Aschenheim, E.: Ist die Eosinophilie ein Symptom der exsudativen Diathese? Zeitschrift für Kinderheilkunde, 1914, Originalien volume 10, page 503.
3. Beck, C.: Die Beteiligung der Schleimhäute des Urogenitalapparates am Symptomenkomplex der exsudativen Diathese. Monatsschrift für Kinderheilkunde, 1913, volume 11, page 468. Reviewed in Jahrbuch für Kinderheilkunde, 1914, volume 78, page 495.
4 v. Behring, E.: Disposition und Diathese. Hamburger medizinische Ueberseehefte, 1914, volume 1, page 2. Reviewed in Zeitschrift für Kinderheilkunde, 1914, Referate, volume 8, page 273.
5. Benfey, A. und Bahrdt, H.: Beitrag zur Beurteilung der Drüsenschwellungen bei Kindern jenseits des Säuglingsalters und ihrer Beziehungen zum Lymphatismus. Zeitschrift für Kinderheilkunde, 1913, Originalien, volume 7, page 481.
6. Bernis, A. L.: Répartition de l'azote urinaire dans quelques dermatoses dites diathésiques. Dissertation Bordeaux 1912.
7. Bloch, I.: Diathesen in der Dermatologie, read at the 28th German Congress for internal medicine, April 19, 1911. Reviewed in Medizinische Klinik, 1911, volume 7, page 752; and Deutsche medizinische Wochenschrift, 1911, volume 38, page 857.

8. Bockhorn, M.: Die exsudativ-lymphatische Diathese und die Prophylaxe. Deutsche medizinische Wochenschrift, 1914, volume 40, page 960.

9. Bouchard: Maladies par ralentissement de la nutrition, Paris, 1882.

10. Bouchard: Traité de pathologie générale, Paris 1900, volume 3.

11. Cameron, H. C.: On diathesis in infancy; a plea for its closer study. British Medical Journal, 1914, page 53. Reviewed in Zeitschrift für Kinderheilkunde, 1914, Referate volume 8, page 512.

12. Czerny, A.: Die exsudative Diathese. Jahrbuch für Kinerheilkunde, 1905, volume 61, page 199.

13. Czerny, A.: Zur Kenntniss der exsudativen Diathese. read at Pediatric Meeting in Dresden, March 23, 1907. Jahrbuch für Kinderheilkunde, 1908, volume 68, page 513.

14. Czerny, A.: Zur Kenntniss der exsudativen Diathese. Monatsschrift für Kinderheilkunde, 1908, volume 7, page 1. Reviewed in Jahrbuch für Kinderheilkunde. 1908, volume 68, page 634.

15. Czerny, A.: Exsudative Diathese, Skrophulose und Tuberkulose. Jahrbuch für Kinderheilkunde, 1909, volume 70, page 529.

16. Czerny, A.: Beitrag zur Leberthrantherapie. Therapie der Gegenwart, 1912, page 49. Reviewed in Zeitschrift für Kinderheilkunde, 1913, volume 3, Referate, page 338.

17. Czerny, A.: Die Bedeutung der Konstitution für die Klinik der kindlichen Infektionskrankheiten. Zeitscrift für ärztliche Fortbildung, 1913, volume 10, page 737. Reviewed in Zeitschrift für Kinderheilkunde, 1914, volume 7, page 354.

18. Comby, J.: Arthritisme. Grancher et Comby, Traité des Maladies de l'Enfance. Paris 1904, volume 1, page 770.

19. Engel: Die Skrophulose und ihre Behandlung. Médizinische Klinik, 1913, volume 9, page 2099.

20. Feer, E.: Ueber exsudative Diathese des Kindes. Korrespondenzblatt für Schweizer Aerzte, 1911, page 676. Reviewed in Zeitschrift für Kinderheilkunde, 1912, Referate, volume 1, page 679.

21. Feer, E.: Das Ekzem mit besonderer Berücksichtigung des Kindesalters. Ergebnisse der inneren Medizin und Kinderheilkunde, 1912, page 316. Reviewed in Zeitschrift für Kinderheilkunde, 1913, Referate, volume 3, page 66.

22. Finkelstein, H.: Zur diätetischen Behandlung des Säuglings und Kinderekzems. Zeitschrift für Kinderheilkunde, 1913, Originalien, volume 8, page 1.
23. Friedjung, J. K.: Die Enährungsstörungen der Brustkinder und Konstitution. Read in Pediatric Section of Gesellschaft für innere Medizin und Kinderheilkunde, Oct. 31, 1912. Reviewed in Zeitschrift für Kinderheilkunde, 1913, Referate, volume 4, page 595.
24. Galup, J.: Le lymphatisme. Diathèse d'anaphylaxie-immunité. Une conception générale des dithèses. Presse médicale 1913, page 313. Reviewed in Zeitschrift für Kinderheilkunde, 1913, Referate, volume 5, page 668.
25. Goilav: Etude sur la bronchite liée à l'herpétisme. Thèse de Paris 1889.
26. Gorter, E.: Ueber exsudative Diathese, Lymphatismus und skrophulőse Konstitution. Geneeskundige Blaaden 1910, volume 5. Reviewed in Jahrbuch für Kinderheilkunde, 1911, volume 73, page 271.
27. de Grandmaison: Traité de l'arthritisme. Paris 1908.
28. Groos, F.: Die Landkartenzunge. Zeitschrift für Ohrenheilkunde und für die Krankheiten der Luftwege, 1913, volume 69, page 26. Reviewed in Zeitschrift für Kinderheilkunde, 1913, Referate, volume 7, page 54.
29. v. Hansemann: Die Konstitution als Grundlage von Krankheiten. Medizinische Klinik, 1912, volume 8, page 933.
30. Heim, P.: Die Konstitutionslehre in der Kinderheilkunde. Berliner klinische Wochenschrift, 1910, page 1784.
31. Helmholz, H.: Eosinophile Blutkőrperchen bei akutem exsudativem Ekzem. Jahrbuch für Kinderheilkunde, 1908, volume 69.
32. Hirschberg, M.: Ekzem und innere Erkrankungen. St. Peterburger medizinische Zeitschrift, 1913, page 313. Reviewed in Zeitschrift für Kinderheilkunde, 1913, Referate, volume 7, page 327.
33. His, W.: Geschichtliches und Diathesen in der inneren Medizin. Read at German Congress for internal medicine, April 19, 1911. Referred in Medizinische Klinik, 1911, volume 7, page 752, and Deutsche Medizische Wochenschrift, 1911, volume 38, page 857.
34. Holt. E.: Diseases of Infancy and Childhood. Fifth edition, New York 1910.
35. Hutinel, V. et Tixier, L.: Arthritisme.—État lymphatique. In V. Hutinel, Les Maladies des Enfants, volume 2, pages 612 and 688, Paris, 1909.
36. Igersheimer: Ueber die Beziehungen von Skrophulose, Lymphatismus, exsudativer Diathese zu den Erkrankungen des Auges. Klinisches Monatsblatt für Augenheilkunde, 1910, page 593. Reviewed in Jahrbuch für Kinderheilkunde, 1911, volume 73, page 271.
37. Jőrg, J. C. G.: Handbuch zum Erkennen und Heilen der Kinderkrankheiten, Leipzig 1826.
38. Kern, H.: Ueber Harnsäureausscheidung bei exsudativen Kindern und ihre Beeinflussung durch Atophan. Jahrbuch für Kinderheilkunde, 1913, volume 78, page 141.
39. Klausner, E.: Ueber lingua geographica hereditaria. Archiv für Dermatologie und Syphilis, 1910, page 103. Reviewed in Jahrbuch für Kinderkrankheiten, 1911, volume 74, page 120.
40. Klotz, M.: Die Bedeutung der Konstitution für die Säuglingsernährung. Würzburger Abhandlungen aus dem Gesamtgebiet der praktischen Medizin, 1911, volume 11, page 181.
41. Krasnogorski, N.: Exsudative Diathese, Vagotonie. Monatsschrift für Kinderheilkunde, 1913, page 129. Reviewed in Jahrbuch für Kinderheilkunde, 1914, volume 78, page 495.
42. Krehl, A.: Presidential Address at the 28th German Congress for Internal Medicine, April 19, 1911. Abstracted in Medizinische Klinik, 1911, volume 7, page 752.
43. Kroll-Lifschütz, A.: Zur Frage der Eosinophilie und exsudativen Diathese. Monatsschrift für Kinderheilkunde 1914, Originalien, volume 12, page 603. Reviewed in Zeitschrift für Kinderheilkunde, 1914, Referate, volume 8, page 291.
44. Lambotte, E.: L'anhydrose et son traitement. Clinique, Bruxelle 1897, volume 11, page 425.
45. Langstein, L. und Rietschel, H.: Kinder und Säuglingskrankheiten. Ergänzungsheft zur Medizinischen Klinik, 1906, volume 2, page 232.
46. Langstein, L.: Erscheinungen von seiten des Magendarmkanals bei exsudativer Diathese. Read at freie Vereinigung für Wissenschaftliche Pädiatrie, March 29,, 1908. Reviewed in Jahrbuch für Kinderheilkunde 1908, volume 67, page 613.
47. Lederer, R.: Exsudative Diathese und Wasserstoffwechsel. Zeitschrift für angewandte Anatomie und Konstitutionsleiden, 1914, volume 1, page 233.
48. Leullier: De l'eczema arthritique chez l'enfant et specialement chez le nourisson. Thèse de Paris 1901.
49. Lévi, A.: L'anaphylaxie dans la pathogénie des états diathésiques de l'arthritisme en particulier. Annales de médicine et chirurgie infantile, 1912, volume 16, page 687. Reviewed in Zeitschrift für Kinderheilkunde 1913, Referate, volume 4, page 601.

50. Lieblein, V.: Zur Kenntniss der lymphatischen Pseudoappendicitis. Wiener klinische Wochenschrift, 1912, volume 25, page 560. Reviewed in Zeitschrift für Kinderheilkunde, 1912, Referate, volume 3, page 637.
51. Lublinski, W.: Ist die Landkartenzunge erblich? Deutsche Medizinische Wochenschrift, 1910, volume 36, page 2343.
52. Lust, F.: Die Beteiligung der Schleimhaut des Urogenitalapparates am Symptomenplex der exsudativen Diathese. Monatsschrift für Kinderheilkunde 1910, volume 10, page 420. Reviewed in Jahrbuch für Kinderheilkunde, 1912, volume 76, page 99.
53. Maillet, F.: Le tissu cellulaire sous-cutané dans la défense de l'organisme de l'enfant. Le Progrès médical, 1912, volume 40, page 81. Reviewed in Zeitschrift für Kinderheilkunde, 1912, Referate, volume 3, page 319.
54. Martius, F.: Pathogenese innerer Krankheiten. Wien 1910.
55. Martius, F.: Konstitution und Vererbung in ihren Beziehungen zur Pathologie. Enzyklopädie der Klinischen Medizin, Berlin, 1914, volume 5.
56. Mautner, F.: Ueber Hautreaktion bei gesunden und ekzematösen Kindern. Zeitschrift Kinderheilkunde, 1913, Originalien, volume 8, page 461.
57. Mendelsohn: Die Frage des Arthritismus. Read at 28th German Congress for internal medicine, April 19, 1911. Abstracted in Medizinische Klinik, 1911, volume 7, page 752, and Deutsche Medizinische Wochenschrift, 1911, volume 38, page 857.
58. Menschikoff, V.: Chlorretention bei exsudativen Prozessen der Haut. Monatsschrift für Kinderheilkunde, 1910, volume 10, page 420. Reviewed in Jahrbuch für Kinderheilkunde, 1912, volume 76, page 99.
59. Méry et Terrien: Die arthritische Diathese im Kindesalter. Ergebnisse der Inneren Medizin und Kinderheilkunde, 1908, volume 2.
60. Merkel: L'asthme chez les enfants. Thése de Paris 1901.
61. de Monchy: Diathesen bei Kindern. Read at meeting of holländische Gesellschaft für Kinderheilkunde, November 26 and 27, 1910. Reviewed in Jahrbuch für Kinderheilkunde, volume 74, page 468.
62. Moro, E. und Kolb, L.: Ueber das Schicksal von Ekzemkindern. Monatsschrift für Kinderheilkunde, 1910, volume 9, page 428.
63. Pässler: Sind die sogenanten Diathesen Konstitutionsanomalien? Münchener Medizinische Wochenschrift, 1913, page 2604. Reviewed in Jahrbuch für Kinderheilkunde, 1914, volume 79, page 500.

64. Pfaundler, M.: Diathesen in der Kinderheilkunde. Read at 28th German Congress for Internal Medicine, April 19, 1911. Abstracted in Medizinische Klinik, 1911, volume 7, page 752, and Deutsche Medizinische Wochschrift, 1911, volume 38, page 857.
65. Pfaundler, M.: Zur Lehre von den kinlichen Diathesen oder Krankheitsbereitschaften. Jahreskurse für ärztliche Fortbildung, Kinderkrankheiten, June 1911. Reviewed in Jahrbuch für Kinderheilkunde, 1911, volume 74, page 486.
66. Pfaundler, M.: Ueber kombinierte Krankheitsbereitschaften oder Diethesen im Kindesalter. Therapie der Gegenwart, 1911, pages 289 and 361. Reviewed in Jahrbuch für Kinderheilkunde, 1911, volume 74, page 601.
67. Pfaundler, M.: Kindliche Krankheitsanlagen (Diathesen) und Wahrscheinlichkeitsberechnung. Zeitschrift für Kinderheilkunde, 1912, volume 4, Originalien, page 175.
68. v. Pfaundler, M.: Besondere Krankheitsbereitschaften (Diathesen) und Konstitutionsanomalien. Feer's Lehrbuch der Kinderheilkunde, 1914, 3rd edition, page 183.
69. v. Planta: Die exsudative Diathese und das hochalpine Gebirgsklima. Korrespondenzblatt für Schweizer Aertze, 1911, page 449. Reviewed in Zeitschrift für Kinderheilkunde, 1912, Referate, volume 1, page 500.
70. Putzig, H.: Das Vorkommen und die klinische Bedeutung der eosinophilen Zellen im Säuglingsalter, besonders bei exsudativer Diathese. Zeitschrift für Kinderheilkunde, 1913, Originalien, volume 9, page 429.
71. Rachford: Lithaemia. Archives of Pediatrics, August, 1897.
72. Rachford: Symptomatology of lithaemia. Archives of Pediatrics, September, 1897.
73. Rachmilewitch: Hautreaktionen von Kindern mit exsudativer Diathese. Jahrbuch für Kinderheilkunde, 1913, volume 77, page 176.
74. Reyher, P.: Konstitution und Säuglingsernährung. Ergebnisse der wissenschaftlichen Medizin, 1911, page 200. Reviewed in Jahrbuch für Kinderheilkunde 1911, volume 74, page 120.
75. Riesel, H.: Adipositas and exsudative Diathese. Zeitschrift für Kinderheilkunde, Originalien, 1911, volume 2, page 325.
76. Rosenstern, J.: Exsudative Diathese und Eosinophilie. Jahrbuch für Kinderheilkunde, 1908, volume 69, page 631.
77. Rozenblat, H.: Scrophulosis, lymphatismus, diathesis exsudativa. Przegl. pedjatr, 1910, No. 3. Reviewed in

Jahrbuch für Kinderheilkunde, 1910, volume 72, page 648.
78. Saenger, M.: Ueber die psychische Komponente unter den Asthmaursachen. Berliner klinische Wochenschrift, 1912, volume 49, page 345. Reviewed in Zeitschrift für Kinderheilkunde, 1912, Referate, volume 3, page 244.
79. Samelson, S.: Die exsudative Diathese. Berlin 1914. Reviewed in Zeitschrift für Kinderheilkunde, 1914, Referate, volume 8, page 463.
80. Schkarin, A.: Ueber Ekzema bei Säuglingen im Anschluss an die Lehre von Diathesen im Kindesalter. Jahrbuch für Kinderheilkunde, 1914, volume 78, page 156.
81. Schlesinger, E.: Das Kőrpergewicht hautkranker, besondere ekzematöser Säuglinge. Read at 9th meeting of West-German Pediatrists. Reviewed in Jahrbuch für Kinderheilkunde, 1908, volume 67, page 467.
82. Schreiber, G.: Les Diathèses infantiles. Archives de médicine des enfants, 1912, volume 15, page 433.
83. Siegert: Die lymphatische Disposition—exsudative Diathese (Czerny) im Säuglingsalter. Münchener medizinische Wochenschrift, 1913, page 1407. Reviewed in Zeitschrift für Kinderheilkunde, 1913, Referate, volume 6, page 177.
84. Simpson, C. A.: Infantile eczema. Journal of the American Medical Association, 1912, volume 58, page 995.
85. Sittler, P.: Die exsudativ-lymphatische Diathese. Würzburg, 1913.
86. Sobelis: Du vomissement périodique chez les enfants. Thèse de Paris, 1889.
87. Spolverini, L. M.: Sulla etiologia e terapia dell'asthma essenziale nei bambini. Rivista di clinica pediatrica, 1913, page 736. Reviewed in Zeitschrift für Kinderheilkunde, 1914, Referate, volume 7, page 736.
88. Steinitz: Ueber Vegetarismus und exsudative Diathese. Read at Pediatric Meeting in Dresden, March 23, 1907. Reported in Jahrbuch für Kinderheilkunde, 1907, volume 65, page 513.
89. Steinitz, F. und Weigert R.: Stoffwechselversuche an Säuglingen mit exsudativer Diathese. Monatsschrift für Kinderheilkunde, 1910, volume 74, page 385.
90. Sthemann: Konstitutionanomalien bei Kindern. Jahrbuch für Kinderheilkunde, 1911, volume 74, page 576.
91. Thiemisch: Aus dem Gebiete der Kinderheilkunde. Beiheft zur Medizinischen Klinik, 1908, volume 4, page 205.
92. Thomson: Guide to the clinical examination and treatment of sick children. London and Edinborough, 1908.

93. Unterberger, S.: Die Bedeutung der Konstitution für den Verlauf der Krankheiten. St. Petersburger medizinische Wochenschrift, 1912, volume 37, page 224. Reviewed in Zeitschrift für Kinderheilkunde, 1912, Referate, volume 3, page 411.
94. Variot, G.: Beobachtungen über die Behandlung des Säuglingsekzems durch Milchwechsel. Reviewed in Jahrbuch für Kinderheilkunde, 1912, volume 75, page 390.
95. Whitmore, A.: Lymphatismus in the East. Lancet 1911, volume 181, page 752. Reviewed in Jahrbuch für Kinderheilkunde, 1912, volume 75, page 390.

481 Franklin Street.

---

**Renal Diabetes.** G. Bergmark, Upsala Läkare förenings Förhandlingar, Jan. 21, reports the case of a woman who showed slight glycosuria but none in the blood. Fasting produced sugar-free urine in the morning, otherwise dietetic measures did not remove the glycosuria. Hyperglycaemia persisted "abnormally long" after giving 100 grams of dextrose. He, therefore considers that the case was not renal but due to some metabolic defect. Of the reported cases of Renal Diabetes, he admits only Gram's case as defensible and is rather inclined to doubt there is such a condition. (Note: It has often occurred to us that the glycogen function, of which we hear so much in physiology and so little in practical medicine, deserves close clinical study and that this might clear up a good many problems in regard to diabetes and glycosuria).

---

**New Blood Stain.** B. Lemchen of Chicago, Med. Rec., April 1, stains and fixes the smear with a saturated solution of benzidin in absolute alcohol for ½ minute. Hydrogen peroxid is then applied for half a minute and the preparation is washed and dried. The red cells, including both protoplasm and nucleus of nucleated reds, are stained blue as are also strands of fibrin if time has been allowed for their formation. White cells and placques do not stain. Obvious deductions are drawn as to the non-relation of the latter to red cells.

---

**Vitamines.** Seidell, Pub Health Reports Feb. 18, has developed a stable vitamine from brewer's yeast, whose use has prevented or cured polyneuritis in pigeons fed on polished rice—i.e. rice mechanically freed from vitamines which are contained almost solely in the seed coats. Pellagra, beri beri, scurvy and possibly rhachitis are diseases ascribed to lack of vitamines.

